# How Different Indicator-Dimension Ratios in Assessment Center Ratings Affect Evidence for Dimension Factors

**DOI:** 10.3389/fpsyg.2020.00459

**Published:** 2020-03-24

**Authors:** Anne Buckett, Jürgen Reiner Becker, Klaus G. Melchers, Gert Roodt

**Affiliations:** ^1^Department of Industrial Psychology and People Management, University of Johannesburg, Johannesburg, South Africa; ^2^Department of Industrial Psychology, University of the Western Cape, Cape Town, South Africa; ^3^Institut für Psychologie und Pädagogik, Universität Ulm, Ulm, Germany

**Keywords:** assessment centers, confirmatory factor analysis, construct-related validity, dimensions, indicator-dimension ratio, item parceling

## Abstract

Previous research on the construct validity of assessment center (AC) ratings has usually struggled to find support for dimension factors as an underlying source of variance of these ratings. Confirmatory factor analysis (CFA) remains the most widely used method to specify and validate the internal structure of AC ratings. However, the research support for dimension effects in AC ratings remains mixed. In addition, competing CFA models (e.g., correlated dimensions-correlated exercises models) are often plagued by non-convergence and estimation problems. Recently, it has been proposed that increasing the number of indicators per dimension and exercise combination might help to find support for dimension factors, in addition to exercise factors, in CFAs of AC ratings. Furthermore, it was also suggested that the increased ratio of indicators to dimensions may also solve some of the methodological problems associated with CFA models used to model AC ratings. However, in this research it remained unclear whether the support for dimension factors was solely due to the use of a larger indicator-dimension ratio or due to parceling that combines several behavioral indicators per dimension and exercise combination into more reliable measures of the targeted dimension. These are important empirical questions that have been left unanswered in the literature but can be potentially meaningful in seeking more balanced support for dimension effects in AC research. Using data from *N* = 213 participants from a 1-day AC, we aimed to investigate the impact of using different indicator-dimension ratios when specifying CFA models of AC ratings. Therefore, we investigated the impact of using different indicator-dimension ratios in the form of item parcels with data from an operational AC. On average, using three parcels eventually led to support for dimension factors in CFAs. However, exercise-based CFA models still performed better than dimension-based models. Thus, the present results point out potential limits concerning the generalizability of recent results that provided support for dimension factors in ACs.

## Introduction

Since their introduction into the workplace, it has been claimed that assessment centers (ACs) provide meaningful evidence of candidates’ on-the-job performance for selection and development purposes (Thornton and Rupp, 2006). In support of this claim, meta-analyses have consistently found support for the criterion-related validity of AC ratings (e.g., Gaugler et al., 1987; Arthur et al., 2003; Krause et al., 2006; Hermelin et al., 2007) and incremental validity over personality and cognitive ability (e.g., Meriac et al., 2008; Sackett et al., 2017). However, despite an illustrious body of predictive, face, and incremental validity evidence (cf. Thornton et al., 2015), researchers have struggled to provide the same level of empirical support for the construct-related validity of AC dimension ratings (e.g., Woehr and Arthur, 2003; Lance et al., 2004b).

As a consequence, ACs have been criticized due to the lack of evidence that AC dimension ratings measure the dimensions that their respective designers were targeting (e.g., Lance, 2008). However, recent research found support for dimension factors in ACs under specific circumstances (Hoffman et al., 2011; Monahan et al., 2013; Kuncel and Sackett, 2014). Specifically, Monahan et al. (2013) found that the use of multiple behavioral indicators per dimension and exercise (i.e., a higher indicator-dimension ratio) eventually led to support for dimension factors – in addition to exercise factors – as a relevant source of variance in AC dimension ratings. On the basis of these results, Monahan et al. (2013) suggested that “the frequent failure to find dimensions in models of the internal structure of ACs is a methodological artifact and that one approach to increase the likelihood for reaching a proper solution is to increase the number of manifest indicators for each dimension factor” (p. 1009).

Even though the results from Monahan et al. (2013) represent an important piece of evidence in support of dimension factors that underlie AC dimension ratings, several relevant questions remained unanswered. Especially, Monahan et al. (2013) did not consider whether the specific number of behavioral indicators allocated to different dimensions influenced support for dimension factors. More knowledge concerning the ratio of behavioral indicators to dimensions in the AC setting is therefore needed for at least two reasons. First, for AC designers and users who are interested in dimension-related information, it would be helpful to know whether there is a specific number of behavioral indicators that are needed per Exercise × Dimension combination to find meaningful evidence for dimension factors. However, the need for a very large number of behavioral indicators may complicate the design of ACs and also exceed the cognitive resources of the assessors who have to observe and evaluate relevant behavior during an AC (cf. Reilly et al., 1990). Thus, even if a high ratio of indicators per dimension would support dimension factors, it may have limited practical value. Second, given that each AC is unique in many respects and also given the long and largely unsuccessful search for support of dimension factors in ACs even when these were designed to measure dimensions, we also considered it as necessary to replicate and extend Monahan et al.’s general pattern of results to see whether their findings do indeed extend to different ACs. Therefore, we wanted to determine whether support for dimension factors can be found in additional samples and, if so, to which degree support for dimension factors is influenced by the use of different indicator-dimension ratios when a similar approach as in the study by Monahan et al. (2013) is followed.

Taken together, the first aim of the current research was to replicate and extend the findings by Monahan et al. (2013). Specifically, for an AC that was designed to capture dimension-relevant information in addition to exercise-specific information we wanted to evaluate whether the use of multiple indicators per dimension and exercise indeed leads to more balanced support for dimension factors in comparison to models with single indicators. Given recent concerns about the replicability of research findings in many different domains (Ioannidis, 2005; Begley and Ellis, 2012; Laws, 2013; American Psychological Society, 2015; Maxwell et al., 2015), this would help to reassure AC designers and practitioners who are targeting dimensions. The second aim was to examine the ratio of indicators to dimensions where evidence of dimension factors becomes visible so as to provide further guidance for AC practitioners.

## Literature Review and Research Questions

### Background on Assessment Centers

The AC is a method that uses behavioral simulation exercises to collect evidence about candidates’ performance on a number of behavioral constructs (most commonly, different performance dimensions; cf. International Taskforce on Assessment Center Guidelines, 2015). Specifically, multiple trained assessors observe candidates across multiple behavioral simulation exercises in a standardized way and evaluate candidates’ performance after each exercise with regard to several different performance dimensions. These collected evaluations are then used as the basis for making selection and/or development decisions. In support of this, as noted above, previous research confirmed the criterion-related validity of AC ratings (Gaugler et al., 1987; Arthur et al., 2003; Hermelin et al., 2007). In addition, there is also evidence that AC ratings can meaningfully improve the prediction of job or training performance beyond other common predictors like cognitive ability and personality (e.g., Meriac et al., 2008; Dilchert and Ones, 2009; Melchers and Annen, 2010; Sackett et al., 2017).

### Perspectives on AC Construct-Related Validity

Historically, dimensions have often been considered the main currency of ACs (Howard, 2008; Arthur, 2012; Thornton and Rupp, 2012). In organizations, dimension ratings further play an essential role in informing human resource practices such as selection, placement, and development. Thereby they serve as a conventional way to report on AC performance (Thornton and Gibbons, 2009). Dimensions have thus enjoyed a prominent role in ACs as the underlying basis for ratings on which employment decisions are made (Rupp et al., 2008). However, previous research has struggled to find support for the construct-related validity of AC dimension ratings (e.g., Lance et al., 2004b; Lance, 2008; Jackson et al., 2016; Wirz et al., 2020).

One premise that complicated matters in AC research in the past pertained to the constituents of legitimate sources of variance in AC ratings (Putka and Hoffman, 2013; Kuncel and Sackett, 2014). Traditionally, variance associated with dimensions was often considered as the primary source of legitimate variance in AC ratings, whereas variance associated with exercises was often considered as reflecting measurement error (Sackett and Dreher, 1982). However, more recently, scholars have argued that exercise factors represent meaningful sources of variance that also contribute to the criterion-related validity of AC ratings (Lance et al., 2000, 2004a, 2007; Jackson, 2012; Putka and Hoffman, 2013).

Furthermore, contemporary approaches in AC research now view the different sources of variance that are present in AC ratings as valuable pieces of information that explain more about candidate performance than dimensions alone (Hoffman, 2012; Hoffman and Meade, 2012; Putka and Hoffman, 2013). Consequently, this *mixed-model* approach acknowledges the interaction between the candidate, exercises, and dimensions (Hoffman, 2012; Melchers et al., 2012). This view is analogous to contemporary views in the personality literature whereby a person’s behavior is examined in relation to his or her interaction with the environment (Funder, 2009). Acknowledging the different sources of variance as in a mixed-model approach is an important turning point for AC research because it suggests that dimension- and exercise-based evidence can be reliably accommodated in feedback, decision-making, and research (Moses, 2008; Borman, 2012).

Even though the mixed-model approach views exercises as well as dimensions as valuable pieces of information, one should keep in mind that the specific evidence that supports the construct-related validity of AC ratings depends on the approach used to design an AC. Specifically, expectations regarding the construct-related validity of AC ratings are informed by one of three main approaches adopted by AC designers. First, when an AC is designed to measure role-based performance aspects that specific to an exercise, then the finding of exercise factors is expected and indicative of construct-related validity (Jackson, 2012). Second, when an AC is designed to measure managerial behavior specific to a set of dimensions, then the expectation would be to find stronger support for dimension-based performance factors (Arthur, 2012). Third, when an AC is designed from the mixed-model perspective, then there would be a reasonable expectation that exercise- as well as dimension-specific aspects of performance should be reflected in a more meaningful way in evaluations of the ACs construct-related validity (Hoffman, 2012).

### Challenges Regarding the Construct-Related Validity of AC Ratings

In order to collect AC ratings for decision-making and research, most commonly, assessors observe candidates in each simulation exercise and then determine scores for each dimension once the exercise has been completed (e.g., Thornton et al., 2015). These scores are known as post-exercise dimension ratings (PEDRs). Although there are other ways to combine and investigate AC ratings (e.g., Kuncel and Sackett, 2014; Wirz et al., 2020), the majority of construct-related validity research used PEDRs as the unit of analysis (Woehr et al., 2012). In addition, confirmatory factor analysis (CFA) remains one of the most popular techniques to use in AC construct-related validity research. But, it is here that research has produced results that are often considered problematic for ACs that are designed with a dimension-based perspective in which AC designers and users target dimension-related information. Specifically, factor analytic studies typically found that most of the variance in PEDR scores is indicative of exercise factors and not of dimension factors. Furthermore, these studies usually have problems to find support for dimension factors in the first place (Lance et al., 2004b; Bowler and Woehr, 2006). This is problematic for ACs that are designed so that dimension ratings in the different exercises should capture similar performance aspects in each exercise.

If, however, one accepts the more contemporary notion that AC performance in many instances represents both dimensions and exercises, then it stands to reason that construct-related validity research should find evidence for, at least, meaningful proportions of variance attributable to dimensions and exercises when an AC is designed accordingly. Nevertheless, this has not consistently been found when PEDRs were used as the unit of analysis in CFAs of AC ratings. Consequently, the support for dimension factors when utilizing PEDRs is equivocal at best, since AC ratings do not always reflect the dimensions they are intended to measure but rather reflect much larger portions of variance attributed to the specific exercises (cf. Sackett and Dreher, 1982; Lance, 2008).

### Recent Developments Concerning the Construct-Related Validity of AC Ratings

It appears that the biggest challenge in previous large-scale analyses of AC datasets (cf. Lance et al., 2004b; Bowler and Woehr, 2006), independent from the specific CFA approach, pertains to issues of convergence and admissibility, which are exacerbated when PEDRs are used as the unit of measurement as they represent single-item measures (Woehr et al., 2012). However, two approaches were recently suggested that seem promising in avoiding the convergence and admissibility problems that have plagued construct-related validity research in the AC domain, and both increase the number of ratings for each postulated dimension factor in the CFAs.

First, in line with the mixed-model approach, by grouping conceptually related dimensions together to form broader dimension categories, Hoffman et al. (2011) were able to find support for latent dimension factors in addition to exercise factors and a general performance factor (GPF), which is conceptually similar to findings of a GPF in job performance ratings (Viswesvaran et al., 2005). In addition, Hoffman et al. also found that these broad dimension factors had incremental criterion-related validity over and above exercises and the GPF (also see Merkulova et al., 2016, for additional evidence). Furthermore, this structure supports contemporary views that candidate behavior also represents situation-specific variance when the exercises are chosen accordingly (Lance et al., 2007) and additionally reflects the general efficacy of candidates when completing management tasks (Thornton et al., 2015).

Although the structure suggested by Hoffman et al. (2011) makes conceptual sense for many ACs, it may be premature to conclude that this approach will resolve empirical challenges in AC construct-related validity research as long as it has not been successfully employed to a broad range of different ACs. Unfortunately, however, research by Siminovsky et al. (2015) revealed limits of this approach. Specifically, they reanalyzed 28 multitrait-multimethod (MTMM) matrices against seven specified CFA models and their analyses returned convergent and admissible solutions in no more than 6 of the 28 matrices analyzed. Moreover, the mean dimension variance was less than 22% of the total variance in the convergent and admissible models. Furthermore, similar to the study by Lance et al. (2004b), the best fitting model was the exercises-only + GPF model, which returned convergent and admissible solutions for 17 of the 28 matrices. This seems at variance with the underlying rationale of ACs that are designed with the intention to measure candidates’ performance on a set of targeted dimensions.

In light of the above discussion, a second related development, and the focus of the current study, was the introduction of adapting the CFA approach by increasing the number of behavioral indicators rated per dimension in an exercise to allow for better model fit of AC data (Monahan et al., 2013). Specifically, in their study, Monahan et al. investigated a condition in which they used neither single PEDRs per exercise and dimension as the unit of analysis, nor multiple conceptually related PEDRs per exercise and broad dimensions. Instead, they used ratings of multiple behavioral indicators per exercise and dimension. Following this methodology, they found that the use of a larger indicator-to-dimension ratio resulted in improved model-data fit and better support for models that included dimension factors. However, in their study, it remained unclear whether the positive results concerning the support of dimension factors depended on the use of a certain number of indicators per dimension. Even though previous research using Monte Carlo simulations suggest that convergence and admissibility rates improve when more than two data points per measurement device and construct combination are available for analyses (Tomás et al., 2000), it is unclear how many data points shall be used in ACs in which support for models with dimension factors is rather limited. Thus, for ACs that are designed with a dimension-based or a mixed-model focus in which dimensions are conceptualized in a way that assumes similar behavioral manifestations across exercises, this leads to the following research question:

**Research Question 1**: What is the ideal indicator-to-dimension ratio to find support for dimension factors?

### Indicator-Dimension Ratios and Parceling of Assessment Center Ratings

In addition to just increasing the indicator-dimension ratio, Monahan et al. (2013) used item parceling in their study. Item parceling is an approach where “two or more items are combined (summed or averaged) prior to an analysis and the parcels (instead of the original items) are used as the manifest indicators of latent constructs” (Little et al., 2013; p. 285). Although item parceling is not without some controversy in the broader literature (see, e.g., Hagtvet and Nasser, 2004; Little et al., 2013; Marsh et al., 2013), most researchers would agree that item parcels go along with several advantages such as less sampling error and thus better indicator reliability, more beneficial distributional characteristics, and fewer dual factor loadings in factor analyses (Bandalos, 2002; Orcan, 2013). As a result of this, higher convergence and admissibility rates of CFA models are found compared to models that use individual items (e.g., Little et al., 2013). Accordingly, the rationale for using parcels in ACs also makes sense because parcels are usually more reliable than single items (e.g., single PEDRs that only represent an overall judgment concerning a given dimension) as they are based on more data points. This suggests that the improved support for dimension factors found by Monahan et al. (2013) may not only be due the increased absolute number of manifest indicators for each dimension factor in the CFA but also to improved measurement properties of these indicators.^1^

With data from two independent samples, Monahan et al. tested various CFA models using common item parceling strategies to examine the role of the indicator-dimension ratio. For the first sample, Monahan et al. formed four item parcels for each Exercise × Dimension combination and randomly assigned behavioral indicators across the different exercise and dimension combinations. Specifically, in this AC six dimensions were targeted across three exercises. For each dimension, assessors had to rate participants’ performance for multiple behavioral indicators (in the form of checklist items) per exercise. Depending on the Exercise × Dimension combination, between 4 and 18 indicators were used per dimension in a given exercise. In Monahan et al.’s second sample, four dimensions were targeted across three exercises. In this AC there were, on average, only three behavioral indicators per Exercise × Dimension combination. Monahan et al. (2013) therefore used each behavioral indicator as a unique data point instead of using the mean across all indicators per dimension as a PEDR and also found support for latent dimension factors. Thus, it seems as if the increased indicator-dimension ratio *per se* was beneficial to find evidence for latent dimension factors. However, at least for their first sample, it might well be possible that the use of parcels also contributed to the beneficial effects with regard to dimension factors.

The current study therefore intends to extend the research of Monahan et al. (2013) by explicitly modeling exercises and dimensions with single indicators as the unit of analysis and comparing the results with CFA models that are specified with parcels as the unit of analysis. This should shed more light on Monahan et al. (2013) findings and leads to our second research question:

**Research Question 2:** Do parcels outperform single indicators during CFAs in finding support for dimension factors in AC ratings?

Taken together, the present article makes at least three contributions to the literature. First, Monahan et al. (2013) set out to show that using more than one data point per Exercise × Dimension combination during analyses (instead of single PEDRs) would lead to evidence of dimension factors in AC ratings for ACs that are targeting dimensions. However, it remained unclear whether there is a specific point at which these dimension effects are more obvious, and the current study explicitly addresses this point. Second, our data is structured in such a way that we have multiple items available for each Exercise × Dimension unit. Therefore, we can determine whether the use of parcels that are built on the basis of multiple behavioral indicators is advantageous in comparison to a condition in which multiple individual indicators are used in terms of model fit, as well as reaching admissible factor analytic solutions. Finally, we tested two additional models that were not considered by Monahan et al. but that can be very informative in AC research, namely correlated dimensions + a general performance factor (CD + GPF) and correlated exercises + a general performance factor (CE + GPF, see below for more information on these models). It remains important to evaluate the impact of increasing the number of indicators per CFA factor and of using parcels on dimension or exercise effects in these two models. The results of the study may guide AC designers who are targeting a GPF in addition to exercises or dimensions in AC ratings.

## Materials and Methods

### Participants

Data were collected from a total of 213 participants of several 1-day ACs. They were supervisors on the same organizational level, working for an energy and chemical manufacturing organization in South Africa. The sample consisted of 66% men and 34% women. The ethnic composition of the sample mainly consisted of black Africans (44%) and whites (37%), followed by Indians (13%), and Coloreds^2^ (6%). The mean age of the participants was 39.06 years (*SD* = 8.08), with a range from 24 to 61 years. Participants completed the AC prior to attending a modular development program for supervisors.

### Assessment Center

The current AC was designed with a mixed-model perspective in mind. Thus, job-relevant situations were used to develop the exercises and a set of dimensions were chosen that should be suitable to be measured in all three exercises, but the measurement of the dimensions was adjusted to the specifics of the exercises.

The AC consisted of three customized simulation exercises designed to measure five specific dimensions, which were identified for development by the organization. The exercises were (a) a role-play exercise dealing with the non-performance of a subordinate, (b) a group exercise consisting of four to six participants working in a team to address five work-related management problems ranging from production problems to people problems, and (c) an in-basket exercise, whereby participants had to deal with a range of emails including a staff scheduling component. These exercises were chosen specifically to represent on-the-job situations that supervisors would deal with on a regular basis in the organization. Furthermore, to improve the realism of the AC, the exercises were designed to be interrelated (i.e., a day-in-the-life of a supervisor).

The AC designers were provided with the competency framework of the organization. The competency framework was created by an established international consulting firm following a strategic realignment process in the organization. In order to select the dimensions for the AC, three criteria were applied. First, dimensions that could be appropriately observed during the AC were identified from the competency framework. Second, the selected dimensions were mapped to the modules targeted for development in the modular development program for supervisors. This was done specifically to ensure that the feedback given to the participants at the end of the AC could be used in a practical way during the development program. Third, a desktop review of the most common jobs at this organizational level was conducted, to ensure that the targeted dimensions were relevant and appropriate. After applying these three criteria, the AC designers identified the five dimensions for inclusion in the AC, which were subsequently approved by the organization.

The dimensions that were targeted during the AC were Business Acumen, Communication, Fostering Relationships, Leadership, and Results Driven. Business Acumen was defined as identifying problems and generating solutions using business knowledge. Communication was defined as conveying information in a clear and coherent way that engages an audience. Fostering Relationships was defined as working well and cooperatively with others to build and maintain effective work relationships. Leadership was defined as using effective interpersonal skills and techniques to provide direction to others to meet objectives and business outcomes. Finally, Results Driven was defined as setting standards for the individual and team and working to meet challenging business goals.

All five dimensions were rated in the role-play and group exercise while three dimensions were rated in the in-basket exercise (Business Acumen, Leadership, and Results Driven). To illustrate the AC approach in the current study, when measuring Business Acumen in the group exercise, an example of a behavioral indicator is “makes logical interpretations based on his/her analysis,” while in the role-play exercise it is “analyzes and interprets information correctly,” and in the in-basket exercise it is “considers key pieces of information and data in analysis of the problem.”

Finally, based on recommendations in the literature, the following features were incorporated into the design of the AC: Only a limited number of dimensions were assessed (Gaugler and Thornton, 1989), assessors received rater training (Lievens, 2001), only expert assessors were used (Kolk et al., 2002), and dimensions were made known to participants given that this AC was used in a development context (Kleinmann et al., 1996; Kolk et al., 2002).

### Procedure

A minimum of 4 and a maximum of 12 participants were assessed in each one-day AC. To assist assessors to score multiple dimensions and exercises for multiple participants in a short timeframe, the structured rating forms for each dimension assessed in an exercise were adjusted to reflect the specifics of the particular exercise. For each Exercise × Dimension combination a range of 7 to 16 indicators were used (see Appendix Table A1). Assessors were instructed to rate each behavioral indicator on a four-point scale where 1 = *Development area*, 2 = *Rounding off*, 3 = *On target*, and 4 = *Strength*. Participants received individual feedback on their AC performance at the end of the day. Data were collected over the course of three years, from 2012 to 2014.

The assessors were psychologists with extensive AC experience. They completed one day of assessor training prior to implementation of the first AC. The training focused on behavior observation and evaluation of the three simulation exercises, in addition to organization-specific information relevant to the AC. The training combined AC behavior observation training with frame-of-reference training (Lievens, 2001). The same group of assessors was used across the three-year period, and several ACs were conducted every year. Assessors underwent recalibration training once a year, to ensure standardization and reliability of scores.

Each assessor observed one participant at a time using the within-exercise scoring approach whereby participants were evaluated at the end of an exercise for each of the targeted dimensions (Thornton et al., 2015). For the group exercise, assessors discussed and calibrated their scores for each participant, but a single assessor was responsible for evaluating the performance of one participant. For the role-play exercise another assessor and the role player (a second assessor) discussed the performance of the participant and arrived at a consensus score for each dimension. The in-basket exercise was scored by a single assessor. Participants were always rated by a different assessor for each exercise and only one assessor was ultimately responsible for evaluating the performance of a given participant in each exercise.

### General Analytic Approach

Data from the current sample contained both item level (i.e., scores for each behavioral indicator) and PEDR information, which allowed us to test for evidence of dimension effects using both single indicators and item parcels in different configurations. Specifically, we wanted to determine whether a particular parcel-to-dimension ratio leads to more meaningful support for dimension factors that underlie AC ratings. We used Mplus 8 (Muthén and Muthén, 1998-2017) to conduct CFAs to investigate both research questions.

For Research Question 1, which concerned the ideal indicator-to-dimension ratio to find support for dimension factors, we used different item parceling combinations prior to conducting the CFAs. A further aspect that is important even with an increased indicator-dimension ratio concerns the psychometric properties of the AC ratings that are collected. Specifically, Brannick (2008) maintains that “construct validity evidence is poor because the exercises are based on tasks sampled for content rather than chosen or designed for illuminating individual differences on the constructs” (p. 131). To this end, he proposed that ACs might be designed to include multiple items within an exercise that are more closely linked to the dimensions. Furthermore, he suggested that more attention should be given to the internal consistency of the measures that represent a given dimension. A means to achieve this end could be to conduct an exploratory factor analysis (EFA) on the multiple indicators for each Dimension × Exercise combination as a first step to get rid of behavioral indicators that do not align with ratings of the other indicators of the same dimension within an exercise. Eventually, this should leave the researcher with the most reliable behavioral indicators to include in further CFAs. Accordingly, we followed Brannick’s (2008) recommendation by omitting problematic items from parcels before specifying and estimating the CFA models. Therefore, we first conducted EFAs across the Exercises × Dimensions (see below for more information) to identify and remove problematic items (e.g., items with very low factor loadings, cross-loaded items, and items with negative factor loadings) before generating results for five conceptualized CFA models. We additionally considered Cronbach’s alphas on the data as an alternative guide of the behavioral indicators to be retained for subsequent analyses. Results from this approach largely corresponded with the results of the EFA insofar as items with low factor loadings also reported low item-total correlations in the item analyses.

In order to compare our findings with those of Monahan et al. (2013), we used the same set of CFA models as that study. These models are also common in most AC construct-related validity research. Model 1 represents the CDCE model that is based on the assumption that both dimensions and exercises are reflected in AC ratings. Thus, this model takes a mixed-model perspective. The correlated dimensions (CD) model (Model 2) has a pure dimension-based perspective and proposed no exercise factors. The correlated exercises (CE) model (Model 3) proposed no dimension factors. In addition, we also tested two other models that were not considered by Monahan et al. (2013) but that are often used in other AC studies: The correlated dimensions + GPF model (CD + GPF; Model 4), which proposes correlated dimension factors and a single general performance factor, and the correlated exercises + GPF model (CE + GPF; Model 5), which proposes exercise factors and a single general performance factor. Furthermore, we allowed dimension factors to be correlated with each other in the models that specified dimension factors, and exercise factors to be correlated with each other in the models that specified exercise factors. However, exercise factors, dimension factors, and the GPF were specified as uncorrelated with each other. Even though this is in line with previous research (e.g., Lance et al., 2004b; Hoffman et al., 2011; Monahan et al., 2013; Merkulova et al., 2016), it might be argued that it makes little conceptual sense that a general performance construct is unrelated to performance on dimension constructs. In this regard, however, we first want to stress that AC dimensions do not represent constructs, and second, that the introduction of additional correlations between the GPF and the dimension and/or the exercise factors would have increased estimation problems.

Accordingly, based on the above models, the EFA approach differed when the models included only Exercises, only Dimensions, and when the models included Exercises × Dimensions. To illustrate, for models including exercises (i.e., Models 3 and 5) the EFA was conducted by including all the dimension ratings in a given exercise. Eigenvalues demonstrated a single factor solution and thus parcels were created for each exercise. For models including dimensions (i.e., Models 2 and 4), the EFA was specified across exercises for the same dimensions. For example, for Business Acumen, we conducted one EFA using all the behavioral indicators across the three exercises. Our results suggested that items clustered together based on the targeted exercise. Comparable results were found for the other dimensions. After the removal of problematic items according to our criteria, parcels were then created per factor. This approach was followed for each of the five dimensions. Finally, for the model including exercises and dimensions (i.e., Model 1), the EFA was conducted for each Exercise × Dimension combination. The final results yielded a total of 28 indicators for Business Acumen, 15 indicators for Communication, 19 indicators for Fostering Relationships, 25 indicators for Leadership, and 26 indicators for Results Driven for further analyses (see Appendix Table A1).

Each of these models was tested across four parceling combinations. This entailed combinations of all the behavioral indicators per Exercise, Dimension, or Exercise × Dimension combination into a single parcel (i.e., ratings of all behavioral indicators were averaged to yield a single PEDR), or combining the ratings of the available indicators per Exercise, Dimension, or Exercise × Dimension combination into two parcels (2P), three parcels (3P), or four parcels (4P). Parcels were formed by randomly assigning behavioral indicators to parcels for each specified model.

For ease of understanding the various approaches discussed thus far, we provide an illustration of what this would look in practice in this study; namely PEDRs, item parcels and single indicator approaches. For example, using the traditional approach of PEDRs either collects only a single post-exercise dimension rating instead of ratings of separate ratings of different items OR it takes the average of the different item ratings as an indicator of the PEDR. We illustrate this approach for a situation in which four behavioral indicators are available for an Exercise × Dimension combination. In the traditional approach the average of all the items (i.e., Item 1 + Item 2 + Item 3 + Item 4) is calculated to get to the PEDR for each Dimension × Exercise combination. Therefore, the PEDR gives us one data point for analyses. Analyses based on such PEDRs usually lead to results that are considered as problematic for dimension construct-related validity by most AC designers.

When using an item parceling approach with the same four behavioral indicators (as a very basic example only), then the average of Item 1 + Item 2 forms Parcel 1; while the average of Item 3 + Item 4 forms Parcel 2. Therefore, with this approach we now have two data points per Dimension × Exercise combination that are available for analyses instead of a single PEDR. The rationale is that using a larger number of data points should improve CFA outcomes which would represent empirical support for conceptually interpreting these ratings in ACs. Furthermore, in comparison to a situation in which only a single overall rating per Exercise × Dimension combination is available, this should reduce sampling error.

Our comparison condition approach uses each behavioral indicator as a data point. Therefore, Item 1 = Single Indicator 1; Item 2 = Single Indicator 2 and so forth. Accordingly, the condition merely uses the individual items as an additional data point during analysis. The purpose of this approach is to test whether it is item parcels that improve results or merely the process of using multiple indicators. This approach therefore specifically allows us to test for this.

For Research Question 2 concerning whether parcels outperform single indicators during CFAs in finding support for dimension factors in AC ratings, we used a selection of single indicator combinations prior to CFA, instead of parcels. In order to specify the competing models with single indicators, the four behavioral indicators with the largest factor loadings in the EFA analyses were used for the CFAs. Indicators were allocated to one of four different configurations, namely one indicator (1I), two indicators (2I), three indicators (3I), or four indicators (4I). For example, for the 2I approach, the two behavioral indicators with the largest factor loadings were used for the CFA. This allowed us to make a head-to-head comparison of a parceling approach when parcels consisted of multiple indicators versus an approach using several single indicators, and to test whether any improvements of construct-related validity were solely a result of increasing the indicator-dimension ratio or also due to the positive manifold produced when using a parceling approach. For comparison purposes, a totally disaggregated CFA model was also specified for each of the five CFA model configurations. This was done to test whether there is better model termination and model fit when using all the behavioral indicators as manifest variables. Thus, the totally disaggregated model tested the idea that using more indicators will be beneficial in general.

Following common guidelines (Bentler, 1990; Cheung and Rensvold, 2002), models were evaluated according to the χ^2^-statistic, the standardized root mean squared residual (SRMR), the root mean squared error of approximation (RMSEA), the Tucker-Lewis index (TLI), the comparative fit index (CFI), and the Akaike information criterion (AIC). In addition to model fit, we also evaluated the various CFA models according to model termination and out-of-bounds estimates.

As noted above, the current AC was designed so that the exercises represented job-relevant managerial situations and the measurement of the same dimensions across exercises was adjusted according to the aim of the exercise. Accordingly, evidence for the construct-related validity of the present AC would require us to find meaningful support for dimension as well as exercise factors, that means, for Model 1.

## Results

Table 1 presents the model termination and model fit indices across the five models (Models 1 to 5) and configurations of single indicators (1I, 2I, 3I, and 4I) when only single indicators were used for analysis. Furthermore, Table 1 contains an additional configuration, (“All Indicators”) to display the results of the totally disaggregated approach in which all the available indicators were used to specify the different models. Similarly, Table 2 presents the model termination and model fit indices across the five models (Models 1 to 5) and the four parceling configurations (PEDR or 1P, 2P, 3P, and 4P) when parcels were created using multiple indicators per Exercise × Dimension combination.

**TABLE 1:** Model-data fit indices for confirmatory factor analysis models for combinations of single indicators.

Model	Model termination	Admissibility	χ^2^	*df*	*p*-Value	SRMR	RMSEA	TLI	CFI	AIC
**Model 1: Correlated dimensions-correlated exercises**										
1 Indicator*	Yes	No	38.69	39	0.48	0.04	0.00	1.00	1.00	142.69
2 Indicators*	No	No								
3 Indicators	No	No								
4 Indicators	No	No								
All Indicators*	Yes	Yes	12912.07	6089	0	0.07	0.07	0.58	0.60	96019.03
**Model 2: Correlated dimensions**										
1 Indicator*	Yes	No	445.10	55	0	0.14	0.18	0.44	0.61	517.10
2 Indicators*	Yes	Yes	1780.02	289	0	0.18	0.16	0.47	0.53	1904.02
3 Indicators*	Yes	Yes	3485.76	692	0	0.19	0.14	0.44	0.48	3661.76
4 Indicators*	Yes	Yes	5239.98	1264	0	0.18	0.12	0.44	0.47	5467.98
All Indicators*	Yes	Yes	18382.41	5875	0	0.15	0.10	0.32	0.34	83114.68
**Model 3: Correlated exercises**										
1 Indicator	Yes	Yes	126.41	62	0	0.06	0.07	0.92	0.94	184.41
2 Indicators	Yes	Yes	758.09	296	0	0.08	0.09	0.84	0.86	868.09
3 Indicators	Yes	Yes	1743.74	699	0	0.08	0.08	0.79	0.81	1905.74
4 Indicators	Yes	Yes	2955.26	1271	0	0.08	0.08	0.77	0.78	3169.26
All Indicators	Yes	Yes	11934.59	5561	0	0.07	0.07	0.58	0.59	92418.41
**Model 4: Correlated dimensions + general performance factor**										
1 Indicator	No	No								
2 Indicators	Yes	Yes	1169.66	263	0	0.13	0.13	0.65	0.72	1345.66
3 Indicators*	Yes	Yes	2445.67	654	0	0.14	0.11	0.62	0.67	2697.67
4 Indicators*	Yes	Yes	3909.61	1212	0	0.13	0.10	0.61	0.64	4241.61
All Indicators*	Yes	Yes	15207.70	5765	0	0.10	0.09	0.48	0.50	80454.15
**Model 5: Correlated exercises + general performance factor**										
1 Indicator	No	No								
2 Indicators	No	No								
3 Indicators	Yes	Yes	1576.05	660	0	0.07	0.08	0.81	0.83	1816.05
4 Indicators	Yes	Yes	2776.59	1219	0	0.08	0.08	0.77	0.79	3094.59
All Indicators	Yes	Yes	9750.83	5454	0	0.07	0.06	0.65	0.67	92042.23

Model termination = converged to a solution; admissibility = no out-of-bounds estimates; PEDR = post-exercise dimension ratings; SRMR = standardized root squared mean residual; RMSEA = root mean squared error of approximation; TLI = Tucker-Lewis index; CFI = comparative fit index; AIC = Akaike information criterion; *Analyses returned a non-positive definite psi matrix.

**TABLE 2:** Model-data fit indices for confirmatory factor analysis models for parcels.

Model	Model termination	Admissibility	χ^2^	*df*	*p*-Value	SRMR	RMSEA	TLI	CFI	AIC
**Model 1: Correlated dimensions-correlated exercises**										
PEDR	Yes	No	70.07	39	0	0.06	0.06	0.97	0.99	200.07
2 Parcels	No	No								
3 Parcels	Yes	Yes	1238.74	650	0	0.06	0.07	0.90	0.91	6234.90
4 Parcels	No	No								
**Model 2: Correlated dimensions**										
PEDR*	Yes	Yes	1321.36	55	0	0.17	0.33	0.17	0.42	3615.55
2 Parcels	Yes	Yes	3178.15	289	0	0.27	0.22	0.33	0.41	5519.34
3 Parcels*	Yes	No	4213.27	692	0	0.22	0.16	0.43	0.47	9265.66
4 Parcels	No	No								
**Model 3: Correlated exercises**										
PEDR	No	No								
2 Parcels*	Yes	No	2.31	6	0.89	0.01	0.00	1.01	1.00	234.83
3 Parcels	Yes	Yes	16.00	24	0.89	0.02	0.00	1.01	1.00	105.08
4 Parcels	No	No								
**Model 4: Correlated dimensions + general performance factor**										
PEDR	No	No								
2 Parcels*	Yes	Yes	1909.65	263	0	0.14	0.17	0.58	0.66	4299.55
3 Parcels*	Yes	Yes	2424.87	653	0	0.14	0.11	0.70	0.73	7556.06
4 Parcels	No	No								
**Model 5: Correlated exercises + general performance factor**										
PEDR	No	No								
2 Parcels	No	No								
3 Parcels	Yes	Yes	5.26	15	0.99	0.00	0.00	1.01	1.00	112.57
4 Parcels	No	No								

Model termination = converged to a solution; admissibility = no out-of-bounds estimates; PEDR = post-exercise dimension ratings; SRMR = standardized root squared mean residual; RMSEA = root mean squared error of approximation; TLI = Tucker-Lewis index; CFI = comparative fit index; AIC = Akaike information criterion; *Analyses returned a non-positive definite psi matrix.

As can be seen in Table 1, in contrast to Monahan et al.’s (2013) Sample 2 that used three indicators for each Exercise × Dimension combination, Model 1 (CDCE) did not converge to an admissible solution, except when all the available indicators were used to specify the different dimensions (and then the fit was rather poor). However, with the exception of the 1I approach in Model 2 (CD) and Model 4 (CD + GPF), all the single indicator configurations converged to proper solutions for Models 2 and 4, even though the fit indices were poor. In Model 3 (CE), all the indicator configurations fit the data well, which was in line with previous findings (Lance et al., 2004b; Monahan et al., 2013). For Model 5 (CE + GPF), only the 3I, 4I and “All Indicators” configurations arrived at an admissible solution, but the fit indices did not represent a good fit.

For Models 2 and 4, all the fit indices represented poor fit independent from the number of indicators. In contrast to this, for Model 3, the 1I approach had the best fit (SRMR = 0.06; RMSEA = 0.07; TLI = 0.92; CFI = 0.94), and for Model 5 the 3I approach had the best fit (SRMR = 0.07; RMSEA = 0.08; TLI = 0.81; CFI = 0.83).

Similar to Monahan et al. (2013), we found support for using combinations of single indicators during analysis for most models. The exceptions were the 1I, 2I, 3I, and 4I configurations for Model 1 (CDCE), the 1I configuration for Model 2 (CD) and Model 4 (CD + GPF), and the 1I and 2I configurations for Model 5 (CE + GPF). Model 3 (CE) had a better fit to the data than Model 2 (CD) when inspecting the fit indices. When inspecting the fit indices for “All Indicators” for each of the five models, the findings were worse in relation to 1I, 2I, 3I and 4I, especially in terms of the TLI and CFI. Therefore, the results show that there is no clear evidence that using more indicators per factor is beneficial to find support for the specified factors.

For the models that used parcels, results are shown in Table 2. As can be seen there, none of the models terminated to an admissible solution for the 4P configurations. Although the PEDR and 2P configuration did not converge to admissible solutions for Model 1 (CDCE), the 3P (SRMR = 0.06; RMSEA = 0.07; TLI = 0.90; CFI = 0.91) configuration converged to a proper solution for this model and represented a good fit. This finding is close to Monahan et al.’s (2013) results for their Sample 2 (SRMR = 0.06; RMSEA = 0.08; TLI = 0.96; CFI = 0.97) and slightly better with respect to the SRMR and RMSEA for the results for their Sample 1 (SRMR = 0.11; RMSEA = 0.10; TLI = 0.94; CFI = 0.95). For Model 2 (CD), in contrast to the other models, the 3P configuration did not arrive at an admissible solution. In addition, all the fit indices for Model 2 represent poor fit independent from the number of parcels. Comparatively, even though the PEDR and 2P configurations arrived at admissible solutions, the fit indices were collectively the worst in comparison to the other models. For Model 3 (CE) the 3P (SRMR = 0.02; RMSEA = 0.00; TLI = 1.01; CFI = 1.00) configuration represented the best fit, but given the near-perfect fit indices, it raises questions about the credibility and replicability of this model in other studies. For Model 4 (CD + GPF) both the 2P and 3P configurations arrived at an admissible solution, with the 3P (SRMR = 0.14; RMSEA = 0.11; TLI = 0.70; CFI = 0.73) configuration performing marginally better than the 2P (SRMR = 0.14; RMSEA = 0.17; TLI = 0.58; CFI = 0.66) configuration. However, neither of the fit indices represent a good fit. For Model 5 (CE + GPF) the 3P (SRMR = 0.00; RMSEA = 0.00; TLI = 1.01; CFI = 1.00) configuration represented the best fit. However, similar to Model 3 (3P) this configuration appeared to be overfitted.

In line with Monahan et al.’s (2013) results, we expected to find a better fit for the 3P and 4P approaches over PEDRs, 2P, and a disaggregated approach across the five models. Although the results did not provide a definitive answer in this regard, when reviewing the fit indices collectively, the 3P approach in Table 2 seemed to fare better, on average, than most other configurations, with the exception of Model 2 (CD). Closer inspection of Table 1 shows that a large number of models containing dimensions returned a non-positive definite psi matrix, even when returning admissible solutions. Because of these non-positive definite psi matrices, these models are not plausible. Conversely, closer inspection of Table 2 shows fewer instances of models returning a non-positive definite psi matrix.

Tables 3 and 4 also show average factor loadings and negative factor loadings for the CFA models using single indicators and parcels, respectively. As can be seen there, Model 3 (CE) led to the highest average factor loadings in comparison to the other models in both Tables 3 and 4. Overall, however, parcels yielded somewhat higher average factor loadings (Table 4) than single indicators (Table 3) and had fewer negative factor loadings.

**TABLE 3:** Summary of model parameters across models for combinations of single indicators.

Model	Lowest estimate	Highest estimate	Average factor loading	Number of negative factor loadings
**Model 1: Correlated dimensions-correlated exercises**				
1 Indicator				
2 Indicators				
3 Indicators				
4 Indicators				
All Indicators	0.00	0.88	0.27	50
**Model 2: Correlated dimensions**				
1 Indicator				
2 Indicators	0.17	0.86	0.46	0
3 Indicators	0.10	0.89	0.46	0
4 Indicators	0.09	0.88	0.45	0
All Indicators	0.01	0.98	0.34	10
**Model 3: Correlated exercises**				
1 Indicator	0.46	0.91	0.67	0
2 Indicators	0.41	0.90	0.69	0
3 Indicators	0.41	0.88	0.68	0
4 Indicators	0.23	0.88	0.66	0
All Indicators	0.01	0.88	0.48	9
**Model 4: Correlated dimensions + general performance factor**				
1 Indicator				
2 Indicators	0.04	0.90	0.39	6
3 Indicators	0.01	0.91	0.31	1
4 Indicators	0.00	0.88	0.34	4
All Indicators	0.00	0.91	0.26	42
**Model 5: Correlated exercises + general performance factor**				
1 Indicator				
2 Indicators				
3 Indicators	0.00	0.86	0.44	9
4 Indicators	0.00	0.95	0.42	3
All Indicators	0.01	0.87	0.30	24

**TABLE 4:** Summary of model parameters across models for parcels.

Model	Lowest estimate	Highest estimate	Average factor loading	Number of negative factor loadings
**Model 1: Correlated dimensions-correlated exercises**				
PEDR				
2 Parcels				
3 Parcels	0.02	0.92	0.47	2
4 Parcels				
**Model 2: Correlated dimensions**				
PEDR	0.04	0.80	0.45	0
2 Parcels	0.15	0.96	0.51	0
3 Parcels	0.10	0.94	0.49	0
4 Parcels				
**Model 3: Correlated exercises**				
PEDR				
2 Parcels	0.18	1.04	0.73	2
3 Parcels	0.21	0.98	0.77	0
4 Parcels				
**Model 4: Correlated dimensions + general performance factor**				
PEDR				
2 Parcels	0.00	0.96	0.40	5
3 Parcels	0.01	0.88	0.38	11
4 Parcels				
**Model 5: Correlated exercises + general performance factor**				
PEDR				
2 Parcels				
3 Parcels	0.16	0.94	0.61	0
4 Parcels				

An unexpected aspect concerned the results for models including a GPF in that neither 1I (Table 1) nor PEDRs (Table 2) converged to admissible solutions for Model 4 (CD + GPF) or Model 5 (CE + GPF). However, all single indicator configurations that returned admissible solutions in Models 4 and 5 also had negative factor loadings (Table 3), while this was not the case for parcels for Model 5 (Table 4). With regard to the parcel configurations, Model 1 (CDCE) showed two negative factor loadings (Table 4) which is a better result than Monahan et al. (2013) who found seven negative factor loadings on the dimension factors in their Sample 1. This is likely due to the EFA pruning strategy used in the current study to eliminate problematic items prior to CFA. However, with the exception of the 3P approach for Model 5 in Table 2, all indicator and parceling approaches yielded poor fit indices overall or returned a non-positive psi matrix. As already noted above, this suggests that these models are therefore also not plausible. Conversely, when comparing Models 4 (CD + GPF) and 5 (CE + GPF), Model 5 performed better overall than Model 4 in terms of model-data fit. Furthermore, when comparing the performance of single indicators versus parcels with Model 5, the 3P approach (Table 2) outperformed all single indicator configurations (Table 1).

In summary, the results seem to suggest that when it comes to estimation stability then single indicators work better than parcels, at least superficially. However, when considering all the available information and when applying our criteria across Tables 1 to 4 (i.e., model fit, convergence, admissibility, number of out-of-bounds issues, and number of non-positive definite psi matrices) then it seems that parcels perform slightly better than single indicators.

## Discussion

The current study aimed to examine whether a higher ratio of indicators-to-dimensions and the use of parcels instead of single indicators would provide better evidence of dimension factors in CFAs, and to determine whether adding more data points to CFAs would improve support for dimension factors in AC ratings. Practically, such an approach seemed promising, because it increases the likelihood of arriving at admissible solutions. This would have been beneficial given that the existing AC literature highlights difficulties in reaching convergent and admissible solutions as a key limitation in construct-related validity research (Lance et al., 2002; Lievens, 2009; Monahan et al., 2013).

Our study makes at least three contributions to the literature. First, our results confirmed that increasing the indicator-to-dimension ratio improved model-data fit during CFAs for certain specified models. Nevertheless, there was still only rather limited support for models including dimension factors. Furthermore, the support with regard to model admissibility and model fit for a model with dimension factors was also limited to a three-parcel configuration. Thus, our study only partially substantiates the replicability and generalizability of previous findings by Monahan et al. (2013). Furthermore, our findings add a caveat to these results from Monahan et al. (2013) that suggested that using multiple indicators per factor when fitting CFA models leads to more support for dimension factors. Thus, our finding contributes to a better understanding of the likely maximum ratio of indicators-to-dimensions that would be beneficial when using a parceling approach to conduct construct-related validity research but also to a better understanding of the limits of such an approach. Nevertheless, given that support for Monahan et al.’s (2013) idea was based on only two samples, we felt it was important to test an additional sample using the item parceling approach suggested by Monahan et al. (2013), to gauge the extent to which the approach could be generalized across different samples.

Second, in our head-to-head comparison of single indicators versus parcels, we found that parcels performed only slightly better overall across the specified CFA models. As such, this finding suggests that – in addition to increasing the indicator-to-dimension ratio – the use of parcels can lead to an improvement in measurement properties, which is beneficial for construct-related validity research. However, the differences between using parcels or items in the CFA configurations were marginal and one could argue that the benefit of using parcels may be offset by the relatively large investment needed to develop a large number of behavioral indicators to combine into parcels.

Third, we expanded on the specified CFA models commonly used in construct-related validity research and investigated two additional models including a general performance factor (GPF). These models were also not previously considered by Monahan et al. (2013). Our findings demonstrated that an exercises-only + GPF model performed better than a dimensions-only + GPF model when a parceling approach was used. This finding therefore adds to the existing literature investigating a GPF as part of the internal structure of AC ratings (Lance et al., 2004b; Siminovsky et al., 2015; Jackson et al., 2016). This is an important finding since previous studies suggest that a GPF explains additional, useful information not explained by narrow dimensions and exercises (Lance et al., 2000, 2004a; Merkulova et al., 2016). Even in these models, using parcels seems to lead to better model fit and model termination.

As expected, based on previous research, models including exercises still performed better than models including dimensions. However, in contrast to the current literature that has struggled to reach admissible solutions for CDCE models (Lance et al., 2004b), when using item parceling the 3P configuration for Model 1 (CDCE) returned an admissible solution similar to the findings of Monahan et al. (2013), but the fit was not as good as in their study. Thus, the question arises why the support for models for dimension factors in our study was considerably weaker in comparison to Monahan et al. (2013).

Given the problems in the present study to find support for models with dimension factors one possibility is that Monahan et al.’s (2013) approach is not as easily replicable and does not generalize as straightforwardly as proponents of dimension-based or mixed-model ACs might have hoped. However, it is difficult to draw final conclusions on this issue until more AC datasets using multiple indicators for each Dimension × Exercise combination are analyzed. Nevertheless, our results at least suggest that a caveat seems necessary concerning the replicability and generalizability of Monahan et al.’s results.

Concerning possible reasons that contributed to the different outcomes in our study, a possible explanation for the differences in comparison to Monahan et al. (2013) could be differences in the rating scales that were used. In the present study, a set of behavioral indicators was first defined for a given dimension and was then modified for each exercise to account for the expected behavioral performance within a certain situation. Where practical, examples of likely performance served as behavioral anchors of less effective to most effective performance for a designated behavioral indicator. These rating scales are likely more cognitively complex to score in comparison to behavioral checklists, which were used in the Monahan et al. study. Furthermore, the large number of indicators that had to be evaluated across the exercises and dimensions in the present study could also have overburdened assessors. On the other hand, the use of behavioral checklists by Monahan et al. (2013) may have attenuated assessor cognitive load thereby allowing for greater congruence of assessor ratings across dimensions (Hennessy et al., 1998).

A second explanation may relate to how assessors scored different participants during the AC. The current AC used the within-exercise scoring approach whereby different assessors were required to observe participants’ performance across the different exercises. Even though such an assessor rotation scheme is quite common (e.g., in the meta-analysis by Lance et al. (2004b), assessor rotation was used in all the AC datasets that were considered) it introduces common rater variance into all the ratings that stem from the same exercise which enhances exercise effects. An alternative would be to have assessors observe the dimensional performance of a participant across all exercises. At least for some studies, such an across-exercise scoring approach has been found to have beneficial effects on the construct-related validity of dimension ratings (e.g., Silverman et al., 1986, but see Melchers et al., 2007). However, such an across-exercise rating approach may lead to problems in applied settings, when an additional assessor is needed for each dimension, and all the assessors have to be present during all exercises in which a given dimension is evaluated.

Concerning the results for models including a GPF, the findings conformed to expected patterns from earlier studies. On the one hand, parcels returned admissible solutions for the 2P and 3P configurations in Model 4 (CD + GPF). However, although this finding may at first seem promising for dimensions in the context of a GPF, the poor fit indices and the fact that these configurations also returned a non-positive definite psi matrix makes this model implausible. On the other hand, the 3P approach in Model 5 (CE + GPF) performed better than all the configurations in Model 4. Therefore, this result aligns with the findings of Siminovsky et al. (2015) who found that an exercises-only + GPF was the best fitting solution for AC ratings in their large-scale study. Additionally, Jackson et al. (2016) found that this model accounted for virtually all reliable variance in AC ratings.

### Practical Implications

Our study offers at least three practical implications for the design of dimension-based and mixed-model ACs. First, our results show that parcels are likely to perform slightly better than single indicators in CFAs that evaluate the underlying structure of AC ratings. Thus, we suggest that multiple indicators per dimension are used for each Exercise × Dimension combination so that parcels can be built. For example, to create a three parcel (3P) configuration, a minimum of six behavioral indicators is needed (i.e., two indicators per parcel). Second, although we advocate for multiple indicators, our results show that there is a limit to the number of parcels required, after which point additional data points become redundant. Third, our findings confirm that even if a dimension-based or mixed-model approach is used, exercises remain a key component that influence AC ratings. This has a practical implication for feedback on AC performance and indicates that both dimensional performance and exercise-specific performance feedback should be given.

### Limitations and Implications for Future Research

Several limitations were present in this study that would benefit from future research. First, this study was based on only one AC sample. Even though one intention of our study was to increase the rather limited database testing Monahan et al.’s idea to use multiple indicators per Dimension × Exercise combination, more research is still needed to further evaluate how using multiple data points and parcels instead of single ratings leads to improvements concerning dimension measurement that are as strong as those suggested by Monahan et al. (2013) or whether the more limited effects found in the present study represent a more appropriate picture of the effects that can be obtained. Additionally, given the problems we encountered with this approach, a possible avenue for future research would be to conduct Monte Carlo simulations to test the impact of using different item parceling configurations on the CFA outcomes.

Second, although the AC in this sample was designed according to accepted standards (International Taskforce on Assessment Center Guidelines, 2015), it is possible that certain design considerations contributed to our findings. For example, despite using a consensus approach to scoring participants in the role-play and group exercises, only single assessor ratings were captured, which may have precipitated exercise-specific ratings. The use of multiple assessors per exercise would have increased the reliability of the ratings which might also have beneficial effects for construct-related validity.

As a third limitation, we found negative factor loadings across all parcel configurations for Model 1 (CDCE) and Model 4 (CD + GPF). Negative factor loadings are not uncommon for CDCE models (Hoffman et al., 2011; Monahan et al., 2013), but were unexpected for Model 4. Future research is needed to investigate the role of a GPF on the occurrence of negative factors loadings in such models. Finally, our study focused only on a higher indicator-dimension ratio and a parceling approach, using a random allocation strategy. We did not take into account the impact of more purposive strategies to create parcels that might be better suited to the treatment of multidimensional data. That is, we do not know whether different parceling strategies recommended in the item parceling literature (see Little et al., 2013) would have led to better support for dimension factors in CFAs. However, given that such allocation strategies are less likely to be used for operational ACs we deemed it more appropriate for the present research to use a more straightforward random allocation strategy. Nevertheless, future research might investigate whether there are any improvements in construct-related validity when more purposive strategies are used to create parcels which would lead to insights concerning the limits of such a strategy.

## Conclusion

Although the findings of the present study support those of Monahan et al. (2013), the overall findings were not conclusive. As such, the robustness of this approach still needs to be confirmed in additional AC settings before it can be generalized. In our head-to-head comparison of two approaches, we found that, collectively, parcels performed only slightly better than single indicators during analyses. Furthermore, our results showed that these positive gains were limited to a three-parcel configuration for mixed-model and exercise-based AC models. This suggests that “more is not always better” when it comes to increasing the indicator-to-dimension ratio in AC ratings. In addition, this approach did not strengthen support for dimension-based models. It may therefore be premature to consider this approach to be the panacea to remedy the construct-related validity challenges for dimension-based ACs when it comes to finding support for dimension factors in AC ratings.

## Data Availability Statement

Information about the dataset used in this study can be obtained from the corresponding author, subject to permission from the client organization.

## Ethics Statements

Written informed consent was obtained from each participant completing the AC. The client organization approved the study and gave consent to use the data. The study complied with the ethical requirements of the University of Johannesburg.

## Author Contributions

AB contributed to research design, data collection, data annotation, data analysis, and writing the first draft of the manuscript. JB contributed to research design and data analysis. AB, JB, and KM equally contributed to read, revise, comment, and approve the submitted manuscript. GR contributed to critical feedback concerning the research design and the manuscript.

## Conflict of Interest

The authors declare that the research was conducted in the absence of any commercial or financial relationships that could be construed as a potential conflict of interest.

## Footnotes

1 We would briefly like to mention that, in contrast to Monahan et al. (2013), the previous research by Hoffman et al. (2011) and Siminovsky et al. (2015) did not consider item parceling.

2 In South Africa, the four main ethnic groups are black Africans, whites, Indians, and Coloreds. These ethnic groups are used for statistical reporting in labor force reviews (Statistics South Africa [SSA], 2016). “Colored” in this context indicates a person of mixed race with one parent who is white and one parent who is black.

## Appendix

**TABLE A1:** Number of behavioral indicators measured in each AC exercise and retained after EFA for further analysis for Model 1.

Dimension	Role play exercise	Group exercise	In-basket exercise	Total behavioral indicators	Total retained after EFA
Business Acumen	8	16	7	31	28
Communication	9	9	Not assessed	18	15
Fostering Relationships	12	14	Not assessed	26	19
Leadership	16	12	7	35	25
Results Driven	11	12	8	31	26

## References

[B1] American Psychological Society (2015). Replication in psychological science. *Psychol. Sci.* 26 1827–1832. 10.1177/0956797615616374 26553013

[B2] Arthur W. Jr. (2012). “Dimension-based assessment centers: theoretical perspectives,” in *The Psychology of Assessment Centers*, eds Jackson D. J. R. Lance C. E. Hoffman B. J. (New York, NY: Routledge), 95–120.

[B3] Arthur W. Jr. Day E. A. McNelly T. L. Edens P. S. (2003). A meta-analysis of the criterion-related validity of assessment center dimensons. *Pers. Psychol.* 56 125–154. 10.1111/j.1744-6570.2003.tb00146.x 18808224

[B4] Bandalos D. L. (2002). The effects of item parceling on goodness-of-fit and parameter estimate bias in structural equation modeling. *Struct. Equ. Model.* 9 78–102. 10.1207/S15328007SEM0901-5

[B5] Begley C. G. Ellis L. M. (2012). Raise standards for preclinical cancer research. *Nature* 483 531–533. 10.1038/483531a 22460880

[B6] Bentler P. M. (1990). Comparative fit indexes in structural models. *Psychol. Bull.* 107 238–246. 10.1037/0033-2909.107.2.238 2320703

[B7] Borman W. C. (2012). “Dimensions, tasks, and mixed models: an analysis of three diverse perspectives on assessment centers,” in *The Psychology of Assessment Centers*, eds Jackson D. J. R. Lance C. E. Hoffman B. J. (New York, NY: Routledge), 309–320.

[B8] Bowler M. C. Woehr D. J. (2006). A meta-analytic evaluation of the impact of dimension and exercise factors on assessment center ratings. *J. Appl. Psychol.* 91 1114–1124. 10.1037/0021-9010.91.5.1114 16953772

[B9] Brannick M. T. (2008). Back to basics of test construction and scoring. *Ind. Organ. Psychol.* 1 131–133. 10.1111/j.1754-9434.2007.00025.x

[B10] Cheung G. W. Rensvold R. B. (2002). Evaluating goodness-of-fit indexes for testing. *Struct. Equ. Model.* 9 233–255. 10.1207/S15328007SEM0902-5

[B11] Dilchert S. Ones D. S. (2009). Assessment center dimensions: Individual differences correlates and meta-analytic incremental validity. *Inter. J. Select. Assess.* 17 254–270. 10.1111/j.1468-2389.2009.00468.x

[B12] Gaugler B. B. Rosenthal D. B. Thornton G. C. III Bentson C. (1987). Meta-analysis of assessment center validity. *J. Appl. Psychol.* 72 493–511. 10.1037/0021-9010.72.3.493

[B13] Gaugler B. B. Thornton G. C. III (1989). Number of assessment center dimensions as a determinant of assessor accuracy. *J. Appl. Psychol.* 74 611–618. 10.1037/0021-9010.74.4.611

[B14] Funder D. C. (2009). Persons, behaviors and situations: an agenda for personality psychology in the postwar era. *J. Res. Pers.* 43 120–126. 10.1016/j.jrp.2008.12.041

[B15] Hagtvet K. A. Nasser F. M. (2004). How well do item parcels represent conceptually defined latent constructs? A two-facet approach. *Struct. Equ. Model.* 11 168–193. 10.1207/s15328007sem1102-2

[B16] Hennessy J. Mabey B. Warr P. (1998). Assessment centre observation procedures: an experimental comparison of traditional, checklist and coding methods. *Intern. J. Select. Assess.* 6 222–231. 10.1111/1468-2389.00093

[B17] Hermelin E. Lievens F. Robertson I. T. (2007). The validity of assessment centers for the prediction of supervisory performance ratings: a meta-analysis. *Inter. J. Select. Assess.* 15 405–411. 10.1111/j.1468-2389.2007.00399.x

[B18] Hoffman B. J. (2012). “Exercises, dimensions and the Battle of Lilliput: evidence for a mixed-model interpretation of assessment center performance,” in *The Psychology of Assessment Centers*, eds Jackson D. J. R. Lance C. E. Hoffman B. J. (New York, NY: Routledge), 281–306.

[B19] Hoffman B. J. Meade A. (2012). Alternate approaches to understanding the psychometric properties of assessment centers: an analysis of the structure and equivalence of exercise ratings. *Int. J. Select. Assess.* 20 82–95. 10.1111/j.1468-2389.2012.00581.x

[B20] Hoffman B. J. Melchers K. G. Blair C. A. Kleinmann M. Ladd R. T. (2011). Exercises and dimensions are the currency of assessment centers. *Pers. Psychol.* 64 351–395. 10.1111/j.1744-6570.2011.01213.x

[B21] Howard A. (2008). Making assessment centers work the way they are supposed to. *Ind. Organ. Psychol.* 1 98–104. 10.1111/j.1754-9434.2007.00018.x

[B22] International Taskforce on Assessment Center Guidelines (2015). Guidelines and ethical considerations for assessment center operations. *J. Manag.* 41 1244–1273. 10.1177/0149206314567780

[B23] Ioannidis J. A. (2005). Contradicted and initially stronger effects in highly cited clinical research. *JAMA* 294 218–228. 10.1001/jama.294.2.218 16014596

[B24] Jackson D. J. R. (2012). “Task-based assessment centers: theoretical perspectives,” in *The Psychology of Assessment Centers*, eds Jackson D. J. R. Lance C. E. Hoffman B. J. (New York, NY: Routledge), 173–189.

[B25] Jackson D. J. R. Michaelides G. Dewberry C. Kim Y.-J. (2016). Everything that you have been told about assessment center ratings is confounded. *J. Appl. Psychol.* 101 976–994. 10.1037/apl0000102 26963079

[B26] Kleinmann M. Kuptsch C. Köller O. (1996). Transparency: a necessary requirement for the construct-related validity of assessment centres. *Appl. Psychol. Inter. Rev.* 45 67–84. 10.1111/j.1464-0597.1996.tb00849.x

[B27] Kolk N. J. Born M. P. Van Der Flier H. Olman J. M. (2002). Assessment center procedures: cognitive load during the observation phase. *Int. J. Select. Assess.* 10 271–277. 10.1111/1468-2389.00217

[B28] Krause D. E. Kersting M. Heggestad E. D. Thornton G. C. III (2006). Incremental validity of assessment center ratings over cognitive ability tests: a study at the executive management level. *Int. J. Select. Assess.* 14 360–371. 10.1111/j.1468-2389.2006.00357.x

[B29] Kuncel N. R. Sackett P. R. (2014). Resolving the assessment center construct validity problem (as we know it). *J. Appl. Psychol.* 99 38–47. 10.1037/a0034147 23957688

[B30] Lance C. E. (2008). Why assessment centers do not work the way they are supposed to. *Ind. Organ. Psychol.* 1 84–97. 10.1111/j.1754-9434.2007.00017.x

[B31] Lance C. E. Foster M. R. Gentry W. A. Thoresen J. D. (2004a). Assessor cognitive processes in an operational assessment center. *J. Appl. Psychol.* 89 22–35. 10.1037/0021-9010.89.1.22 14769118

[B32] Lance C. E. Foster M. R. Nemeth Y. M. Gentry W. A. Drollinger S. (2007). Extending the nomological network of assessment center construct validity: prediction of cross-situationally consistent and specific aspects of assessment center performance. *Hum. Perform.* 20 345–362. 10.1080/08959280701522031

[B33] Lance C. E. Lambert T. A. Gewin A. G. Lievens F. Conway J. M. (2004b). Revised estimates of dimension and exercise variance components in assessment center postexercise dimension ratings. *J. Appl. Psychol.* 89 377–385. 10.1037/0021-9010.89.2.377 15065983

[B34] Lance C. E. Newbolt W. H. Gatewood R. D. Foster M. R. French N. R. Smith D. E. (2000). Assessment center exercise factors represent cross-situational specificity, not method bias. *Hum. Perform.* 13 323–353. 10.1207/s15327043hup1304_1

[B35] Lance C. E. Noble C. L. Scullen S. E. (2002). A critique of the correlated trait-correlated method and correlated uniqueness models for multitrait-multimethod data. *Psychol. Methods* 7 228–224. 10.1037//1082-989X.7.2.228 12090412

[B36] Laws K. R. (2013). Negativland - a home for all findings in psychology. *BMC Psychol.* 1:2. 10.1186/2050-7283-1-2 25566354 PMC4269995

[B37] Lievens F. (2001). Assessor training strategies and their effects on accuracy, interrater reliability, and discriminant validity. *J. Appl. Psychol.* 86 255–264. 10.1037/0021-9010.86.2.255 11393438

[B38] Lievens F. (2009). Assessment centers: a tale about dimensions, exercises, and dancing bears. *Eur. J. Work Organ. Psychol.* 18 102–121. 10.1080/13594320802058997

[B39] Little T. D. Rhemtulla M. Gibson K. Schoemann A. M. (2013). Why the items versus parcels controversy needn’t be one. *Psychol. Methods* 18 285–300. 10.1037/a0033266 23834418 PMC3909043

[B40] Marsh H. W. Lüdtke O. Nagengast B. Morin A. J. von Davier M. (2013). Why item parcels are (almost) never appropriate: two wrongs do not make a right - Camoflaging misspecification with item parcels in CFA models. *Psychol. Methods* 18 257–284. 10.1037/a0032773 23834417

[B41] Maxwell S. E. Lau M. Y. Howard G. S. (2015). Is psychology suffering from a replication crisis? *Am. Psychol.* 70 487–498. 10.1037/a0039400 26348332

[B42] Melchers K. G. Annen H. (2010). Officer selection for the Swiss armed forces: an evaluation of validity and fairness issues. *Swiss J. Psychol.* 69 105–115. 10.1024/1421-0185/a000012

[B43] Melchers K. G. Henggeler C. Kleinmann M. (2007). Do within-dimension ratings in assessment centers really lead to improved construct validity? A meta-analytic reassessment. *Zeitschr. Personalpsychol.* 6 141–149. 10.1026/1617-6391.6.4.141

[B44] Melchers K. G. Wirz A. Kleinmann M. (2012). “Dimensions and exercises: theoretical background of mixed-model assessment centers,” in *The Psychology of Assessment Centers*, eds Jackson D. J. R. Lance C. E. Hoffman B. J. (New York, NY: Routledge), 237–254.

[B45] Meriac J. P. Hoffman B. J. Woehr D. J. Fleischer M. S. (2008). Further evidence for the validity of assessment center dimensions: a meta-analysis of incremental criterion-related validity of dimension ratings. *J. Appl. Psychol.* 93 1042–1052. 10.1037/0021-9010.93.5.1042 18808224

[B46] Merkulova N. Melchers K. G. Kleinmann M. Annen H. Szvircsev Tresch T. (2016). A test of the generalizability of a recently suggested conceptual model for assessment center ratings. *Hum. Perform.* 29 226–250. 10.1080/08959285.2016.1160093

[B47] Monahan E. L. Hoffman B. J. Lance C. E. Jackson D. J. R. Foster M. R. (2013). Now you see them, now you do not: the influence of indicator-factor ratio on support for assessment center dimensions. *Pers. Psychol.* 66 1009–1047. 10.1111/peps.12049

[B48] Moses J. (2008). Assessment centers work, but for different reasons. *Ind. Organ. Psychol.* 1 134–136. 10.1111/j.1754-9434.2007.00026.x

[B49] Muthén L. K. Muthén B. O. (1998-2017). *Mplus Users’ Guide*, 7th Edn, Los Angelos, CA: Muthén and Muthén.

[B50] Orcan F. (2013). *Use of Item Parceling in Structual Equation Modeling with Missing Data.* Doctoral Thesis, Florida State University Libraries, Tallahassee, FL.

[B51] Putka D. J. Hoffman B. J. (2013). Clarifying the contribution of assessee-, dimension-, exercise-, and assessor-related effects to reliable and unreliable variance in assessment center ratings. *J. Appl. Psychol.* 98 114–133. 10.1037/a0030887 23244226

[B52] Reilly R. R. Henry S. Smither J. (1990). An examination of the effects of using behavior checklists on the construct validity of assessment center dimensions. *Pers. Psychol.* 43 71–84. 10.1111/j.1744-6570.1990.tb02006.x

[B53] Rupp D. E. Thornton G. C. III Gibbons A. M. (2008). The construct validity of the assessment center method and usefulness of dimensions as focal constructs. *Ind. Organ. Psychol.* 1 116–120. 10.1111/j.1754-9434.2007.00021.x

[B54] Sackett P. R. Dreher G. F. (1982). Constructs and assessment center dimensions: some troubling findings. *J. Appl. Psychol.* 67 401–410. 10.1037/0021-9010.67.4.401

[B55] Sackett P. R. Shewach O. R. Keiser H. N. (2017). Assessment centers versus cognitive ability tests: challenging the conventional wisdom on criterion-related validity. *J. Appl. Psychol.* 102 1435–1447. 10.1037/ap10000236 28530416

[B56] Silverman W. H. Dalessio A. Woods S. B. Johnson R. L. (1986). Influence of assessment center methods on assessor ratings. *Pers. Psychol.* 39 565–578. 10.1111/j.1744-6570.1986.tb00953.x 31339363

[B57] Siminovsky A. B. Hoffman B. J. Lance C. E. (2015). Revised estimates of general performance effects on AC ratings. *Paper Presented at the 30th Annual Conference of the Society for Industrial and Organizational Psychology*, Philadelphia, PA.

[B58] Statistics South Africa [SSA] (2016). *Quarterly Labour Force Survey.* Available at: http://www.statssa.gov.za/publications/P0211/P02111stQuarter2016.pdf (accessed March 10, 2017).

[B59] Thornton G. C. III Gibbons A. M. (2009). Validity of assessment centers for personnel selection. *Hum. Resour. Manag. Rev.* 19 169–187. 10.1016/j.hrmr.2009.02.002

[B60] Thornton G. C. III Rupp D. E. (2006). *Assessment Centers in Human Resource Management: Strategies for Prediction, Diagnosis, and Development.* New York, NY: Lawrence Erlbaum Associates.

[B61] Thornton G. C. III Rupp D. E. (2012). “Research into dimension-based assessment centers,” in *The Psychology of Assessment Centers*, eds Jackson D. J. R. Lance C. E. Hoffman B. J. (New York, NY: Routledge), 141–170.

[B62] Thornton G. C. III Rupp D. E. Hoffman B. (2015). *Assessment Center Perspectives for Talent Management Strategies*, 2nd Edn, New York, NY: Routledge.

[B63] Tomás J. M. Hontangas P. M. Oliver A. (2000). Linear confirmatory factor models to evaluate multitrait-multimethod matrices: the effects of number of indicators and correlation among methods. *Multiv. Behav. Res.* 35 469–499. 10.1207/S15327906MBR3504_03 26811201

[B64] Viswesvaran C. Schmidt F. L. Ones D. S. (2005). Is there a general factor in ratings of job performance? A meta-analytic framework for disentangling substantive and error influences. *J. Appl. Psychol.* 90 108–131. 10.1037/0021-9010.90.1.108 15641893

[B65] Wirz A. Melchers K. G. Kleinmann M. Lievens F. Annen H. Blum U. (2020). Do overall dimension ratings from assessment centers show external construct-related validity? *Eur. J. Work Organ. Psychol.* 10.1080/1359432X.2020.1714593

[B66] Woehr D. J. Arthur W. Jr. (2003). The construct-related validity of assessment center ratings: a review and meta-analysis of the role of methodological factors. *J. Manag.* 29 231–258. 10.1177/014920630302900206

[B67] Woehr D. J. Meriac J. P. Bowler M. C. (2012). “Methods and data analysis of assessment centers,” in *The Psychology of Assessment Centers*, eds Jackson D. J. R. Lance C. E. Hoffman B. J. (New York, NY: Routledge), 45–67.

